# Using the Person-Based Approach to Co-Create and Optimize an App-Based Intervention to Support Better Sleep for Adolescents in the United Kingdom: Mixed Methods Study

**DOI:** 10.2196/63341

**Published:** 2024-10-31

**Authors:** Sarah E Bennett, Milly H Johnston, Georgia Treneman-Evans, James Denison-Day, Anthony Duffy, Amberly Brigden, Paula Kuberka, Nicholas Christoforou, Lee Ritterband, Jewel Koh, Robert Meadows, Doaa Alamoudi, Ian Nabney, Lucy Yardley

**Affiliations:** 1 School of Psychological Science University of Bristol Bristol United Kingdom; 2 Bristol Medical School University of Bristol Bristol United Kingdom; 3 School of Psychology University of Southampton Southampton United Kingdom; 4 Digital Health Circle Lab School of Interactive Arts & Technology Simon Fraser University British Columbia, BC Canada; 5 School of Engineering, Mathematics and Technology University of Bristol, Bristol United Kingdom; 6 Psychiatry and Neurobehavioral Sciences University of Virginia Health System Charlottesville, VA United States; 7 Department of Sociology University of Surrey Guildford United Kingdom; 8 Department of Computer Science University of Bristol Bristol United Kingdom

**Keywords:** behavior change, digital intervention, insomnia, depression, anxiety, sleep, qualitative research, mobile phone

## Abstract

**Background:**

Poor sleep is a common problem in adolescents aged 14 to 18 years. Difficulties with sleep have been found to have a bidirectional link to mental health problems.

**Objective:**

This new research sought to involve young people in the co-creation of a new app, particularly those from underserved communities. The Sleep Solved app uses science-based advice to improve sleep-related behaviors and well-being. The app was developed using the person-based approach, underpinned by the social cognitive theory and the social-ecological model of sleep health.

**Methods:**

Young people (aged 14-18 y) were recruited from across the United Kingdom to contribute to patient and public involvement (PPI) activities. In partnership with our peer researcher (MHJ), we used a multitude of methods to engage with PPI contributors, including web-based workshops, surveys, think-aloud interviews, focus groups, and app beta testing.

**Results:**

A total of 85 young people provided PPI feedback: 54 (64%) young women, 27 (32%) young men, 2 (2%) genderfluid people, 1 (1%) nonbinary person, and 1 (1%) who reported “prefer not to say.” Their levels of deprivation ranged from among the 40% most deprived to the 20% least deprived areas. Most had self-identified sleep problems, ranging from 2 to 3 times per week to >4 times per week. Attitudes toward the app were positive, with praise for its usability and use of science-based yet accessible information. Think-aloud interviews and a focus group identified a range of elements that may influence the use of the app, including the need to pay attention to language choices and readability. User experiences in the form of narrated audio clips were used to normalize sleep problems and provide examples of how the app had helped these users.

**Conclusions:**

Young people were interested in using an app to better support their sleep and mental health. The app was co-created with strong links to theory- and evidence-based sleep hygiene behaviors. Future work to establish the effectiveness of the intervention, perhaps in a randomized controlled trial, would provide support for potential UK-wide rollout.

## Introduction

### Background

Poor sleep is a common issue among adolescents, with significant effects on school performance [1], mood, and behavior [2,3]. Since the 1990s, the amount of sleep per night that adolescents self-report has decreased significantly [4,5]. Poor sleep can impact adolescents’ mental health [6]; reduced sleep duration has been associated with loneliness, low mood [7], and depression [8] and can affect adolescents’ physical health over time [9].

Adolescence is a period of significant biological and psychosocial changes. These changes include a shift in the sleep-wake cycle, a change in the circadian rhythm [10], and a gradual behavioral shift toward later bedtimes [11-13]. The shift toward later bedtimes is more noticeable on school nights compared to weekends, with later sleep times making it more difficult for adolescents to wake up when they need to [12,13]. Rather than later bedtimes, adolescents have been shown to sleep better when sticking to a regular bedtime routine with consistent sleep and wake times [14].

Sleep hygiene, which can be defined as behavioral practices that promote good sleep, plays a key role in adolescents’ sleep quality. Differences in sleep hygiene behavior may partly mediate the relationship between sociodemographic factors and sleep health [15]. Across the wealth of sleep hygiene literature, 3 sleep hygiene behaviors are reported as having a key impact on sleep: avoiding behavioral and cognitive arousal before bedtime, spending less time in bed, and setting a regular wake time [16].

Diet may also play a role in adolescents’ sleep [17,18]. The UK National Diet and Nutrition Survey from 2016 to 2019 found that those aged 11 to 18 years had the highest consumption of sugar-sweetened soft drinks and mean saturated fat intake [19]. Diets high in fat and sugar have been associated with shorter sleep duration [20] and significantly higher prevalence of sleep disturbance in adolescents [21] compared to a healthier diet. A high caffeine intake has been associated with sleep problems, including later bedtimes, shorter sleep duration [22,23], difficulty sleeping, and morning tiredness [24]. Dietary influences on sleep may disproportionately affect adolescents from lower-income households. A descriptive analysis of UK survey data from 2005, 2009, and 2014 found that adolescents aged 11 to 15 years from families with lower levels of affluence reported fewer healthy eating behaviors, higher sugary drink consumption, and lower intake of fruits and vegetables [25].

Previous research has indicated that adolescents from more socioeconomically deprived neighborhoods typically experience poorer sleep quality, a shorter sleep duration, and increased daytime sleepiness [26]. They also have lower sleep efficiency, spending more time awake at night [27]. Possible causes may include more chaotic, noisy [28,29], or crowded living environments with lower neighborhood safety, higher levels of crime, or concerns about violence [27,30] when compared to adolescents from higher-income homes [9,31]. Therefore, adolescents from areas of higher socioeconomic deprivation are more likely to experience poorer-quality sleep.

There is a bidirectional relationship between insomnia and mental health difficulties [32-35]. Anxiety may perpetuate and worsen insomnia, with those who experience emotional dysregulation in childhood being more likely to experience anxiety disorders in adolescence [36-38]. As poor sleep during adolescence may be a precursor to mental health problems, targeting poor sleep behaviors early may prevent the development of mental health difficulties, such as anxiety or depression, at a later stage [32].

However, engaging with adolescents at an early stage can be a challenge. There is evidence suggesting that adolescents are less likely to engage with mental health support services due to perceived stigma, embarrassment [39], or a preference for less formal advice and support [40-44]. Instead, smartphones can be a key route to engaging with adolescents; a range of apps to support adolescents with their sleep and well-being have been designed. Delivering Online Zzz’s With Empirical Support (DOZE), a 4-week intervention, was developed in co-creation with Canadian adolescents [45]. This intervention includes the ability to track sleep time, average time to fall asleep, and nighttime awakening. A “tips” section provides education to users on how best to make changes to their sleep, such as winding down before bed [45]. Similarly, Sleep Ninja was developed in partnership with Australian adolescents through a series of focus groups focusing on the app design [46,47]. The app features 6 educational sessions, a sleep-tracking function, sleep tips, and reminders to wind down in the evening [46]. Finally, the Sleepio app uses cognitive behavioral therapy for insomnia (CBTi) in 6 web-based, 20-minute sessions [48]. Sleepio has shown promising results in a small population of adolescents (N=39) with mental health problems [48]. However, no apps to date have been co-created with adolescents from the United Kingdom, and none have been co-created with those from disadvantaged backgrounds.

The apps described previously are all intended to provide in-depth support through extended use (4-6 sessions). However, there is some evidence that, for many adults and adolescents, a single session of sleep hygiene advice may be sufficient to improve sleep [49]. Our study aim was to design a highly accessible app suitable for adolescents with lower literacy levels that did not require extensive engagement for users to benefit from sleep hygiene advice and support. The purpose of this app was to serve as an accessible first step of a stepped program of support [50], which would guide adolescents with more persistent and serious sleep problems toward the in-depth support provided by existing apps (specifically, Sleep Healthy Using the Internet [SHUTi] [51,52]).

### The Goal of This Study

This paper details the co-creation and optimization of a novel, brief, stepped-care intervention to support adolescents with sleep problems (Table 1) from its initial design phases to co-creation with adolescents from a range of collaborative youth organizations, charities, and educational partners. After outlining the development process, the feasibility of the app and feedback in light of real-world testing are explored. The process was iterative, and contributor-suggested amendments were used to make changes to the intervention as the co-creation process progressed.

**Table 1:** The 2 stages of Sleep Well.

Intervention stage and app		Details	Time when it was offered
**Stage 1**			
	Sleep Solved	A web-based application co-created with adolescents to provide brief (six 5- to 10-min sections) and accessible advice and behavior change support to help improve their sleep. The short Sleep Solved intervention may be sufficient as an intervention for less serious sleep problems.	At sign-up (for iOS and Android users)
	Phone Downtime	An Android app created by a University of Bristol PhD student before this study. Phone Downtime allows users to set their desired wake and sleep times to support their sleep schedule while using the Sleep Solved app.	At sign-up (for Android users only)
**Stage 2**			
	SHUTi^a^	A six 20-min–session program to treat insomnia based on internet-delivered cognitive behavioral therapy was adapted from a version of SHUTi shown to be effective for adults in the United States [51,52]. The content was coadapted in this study to better suit adolescents in the United Kingdom.	Bite Back [53] and SHUTi [51] are offered if adolescents score highly on measures of anxiety and depression (RCADS^b^) or insomnia (Insomnia Severity Index) after 6 weeks of access to Sleep Solved.
	Bite Back	Developed by researchers in Australia, Bite Back [53] is a web-based 6-session positive psychology program designed to improve well-being and resilience in adolescents.	Bite Back [53] and SHUTi [51] are offered if adolescents score highly on measures of anxiety and depression (RCADS) or insomnia (Insomnia Severity Index) after 6 weeks of access to Sleep Solved.

^a^SHUTi: Sleep Healthy Using the Internet.

^b^RCADS: Revised Child Anxiety and Depression Scale.

**Figure 1:**
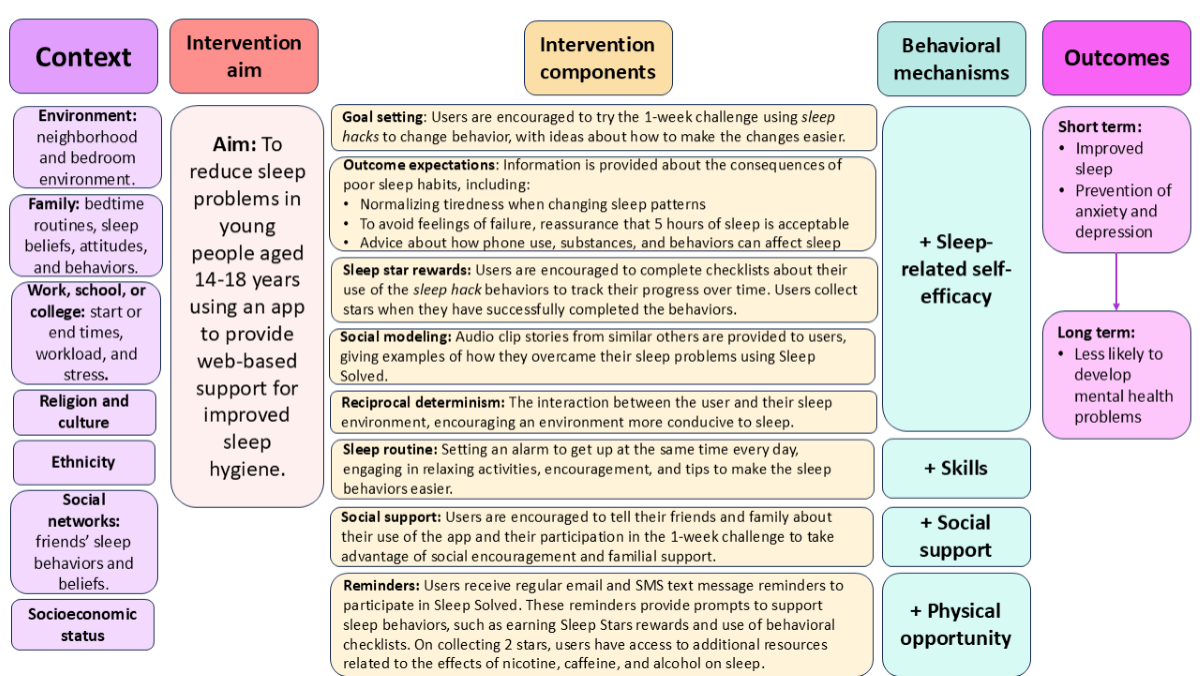
The Sleep Solved logic model.

The person-based approach was used in the development of the Sleep Solved intervention to provide adolescents with a theoretically informed app that was engaging, clear, and appropriate for their needs [54,55]. This involved initially drawing on clinical theory and evidence to specify the core required intervention elements to support behaviors that would help adolescents sleep better, summarized in a logic model (Figure 1). The social cognitive theory by Bandura [56] was used to underpin the behavioral elements of Sleep Solved, whereas the social-ecological model of sleep health [57] was chosen to represent the multifactorial nature of various moderators on sleep and sleep-related interventions at the individual, social, and societal levels, such as the influence of social networks or family [57]. The logic model features 8 intervention components that make up the key content of the app. How these components relate to social cognitive theory, moderators from the social-ecological model of sleep, and the underpinning behavioral mechanisms is outlined in the following paragraphs.

We drew on theory and evidence to create guiding principles (Table 2) that were intended to encourage engagement with Sleep Solved. The coproduction work described in this section was then used to co-design the app content and format. Details regarding the virtual co-design of the design aspects of the intervention have been published separately (A Duffy, unpublished data, May 2022).

**Table 2:** Guiding principles for Sleep Solved.

User characteristics	Design objective	Features to address the objective
Very low tolerance for engaging with advice that is obvious, dull, not credible, or irrelevant to their sleep context Low patience for engaging with apps that are dull, uninteresting, unfriendly, patronizing, or misleading or have a confusing user experience and intrusive advertising	To ensure that all advice is immediately engaging and viewed as interesting, trustworthy, and relevant To ensure that the app itself is designed to be engaging, friendly, entertaining, and motivating and hold the user’s interest and attention, with no advertising	Present novel content in the most concise and engaging format possible Simple, short sentences Good functionality; consistent navigation, theme, gestural design, and user flow Optimized for mobile use Provide evidence of credibility and trustworthiness (eg, team expertise and scientific validity of the advice) Focus on core principles of sleep relevant to a wide range of situations Personalized using relatable stories from other adolescents with sleep problems Gamification—users can collect stars to encourage engagement with the app
Great diversity in needs and preferences for how advice is presented, in accordance with users’ social identity and levels of (health) literacy	To ensure that the advice is appropriate for people with different identities, social contexts, and learning preferences; variety of text, images, and audio clips to reflect different learning styles Simple language, short sentences, and use of images or icons may be more engaging Consistent navigation structure; similar graphics, gestural designs, and theme throughout the app for consistency and ease of use Sleep tips throughout	Ensure that role models and stories are relevant to diverse contexts and identities Present advice in a variety of accessible formats Provide links to a wider range of additional resources or formats (eg, relaxing music, mindfulness, and in-depth resources) Include a summary of useful hints and tips to improve sleep
The importance of choice or free will as opposed to paternalistic advice	Choice architecture—framing advice as an option; emphasizing the benefits of the desired behavior vs the consequences of the undesired behavior	Avoid telling adolescents what they should do (eg, avoid giving a prescribed amount of sleep that adolescents should be getting) to reduce feelings of anxiety and inadequacy in the target population (aged 14-18 years) Present facts, actions, and consequences Frequently asked question pop-ups allowing for more information if required
A preference for web-, app-, or technology-based interactions over face-to-face	App use involves interactions with mobile technology rather than face-to-face interactions	Adolescents using apps may prefer app- and technology-based interactions

Advising adolescents to improve their sleep using a smartphone device known to disrupt sleep may seem counterintuitive, particularly as the nighttime use of smartphones and social media has been linked to disturbed sleep and poor academic achievement [58-61]. However, apps are a key method for engaging adolescents; 81% of adolescents use the internet to seek health-related information [62], whereas digital health interventions to support people with their sleep are flourishing [63]. To avoid too much phone exposure too close to bedtime, users were encouraged to use the app to decide on their preferred sleep behaviors and environment ahead of time, earlier in the evening. Later sections of Sleep Solved remind users that looking at a bright phone screen too close to bedtime could affect their ability to get to sleep [64].

## Methods

### Phase 1: Initial Intervention Development

#### Sleep Solved Ideas and Concept Generation

To explore adolescents’ views and experiences of sleep and well-being, a web-based focus group was conducted with the Bristol Young People’s Advisory Group (YPAG). Patient and public involvement (PPI) contributors were presented with initial ideas for key intervention concepts (eg, a focus on how “science-based” research could help them sleep better and why spending less time in bed could help). Contributors were asked what they thought of these concepts and were invited to share additional ideas for supporting their sleep and well-being.

#### Sleep Solved Ideas: Name and Design

To involve PPI contributors who preferred to provide written feedback, a Qualtrics survey (Qualtrics) was used to elicit feedback regarding the look and feel of Sleep Solved. PPI contributors were asked for feedback via closed- and open-ended questions and to share ideas about the app’s name, logo, color palette, and theme as well as the study’s recruitment materials:

- Potential app names: preferred name from 7 options—*Sleep Solved, Snooze It, Science2Sleep, Slumber Master, Rest Pro, Sleep Tracker,* and *Awake to Sleep*
- Color palettes: three options—(1) crimson red, roundel blue, international orange, and black; (2) black, opaline green, deep cream, and pale roundel blue; and (3) Oxford blue, light orange, deep cream, and ruby
- Logos: from a range of 11 possible logo designs
- Design themes: a total of 6 options from other youth-oriented media

#### New Theme Designs for Sleep Solved

On the basis of the feedback from the first Qualtrics survey, our design partners at PIP Creative designed 2 tailored themes to give the Sleep Solved app a consistent look and feel. PPI contributors were invited via a second Qualtrics survey to rank how much they liked the themes (0-100) and share their thoughts and suggestions regarding the visual design.

#### New Logo Designs for Sleep Solved

On the basis of the feedback from the first Qualtrics survey, 4 viable app logos were developed by PIP Creative. These logos were presented to PPI contributors via a third Qualtrics survey, and contributors were requested to rank their preferred logos. Comments about the logos were encouraged using open-ended questions.

#### Sleep Well Design Feedback

To determine the preferred timing of offering the 3 components of Sleep Well (Phone Downtime, Sleep Solved, and SHUTi [51]), 3 different pathway options were presented to adolescents in a fourth Qualtrics survey. For each pathway, PPI contributors were invited to share how likely they were to take part in the Sleep Well study for 6 weeks, giving a rating from 0 (“not at all”) to 100 (“a lot”) and their reasons why.

### PPI Contributors

Adolescents aged 14 to 18 years were recruited to co-design the intervention through partnerships with national educational groups and charities: the Association of Colleges, the E-ACT multiacademy trust, the McPin Foundation, the Bristol YPAG, and Off The Record Bristol. We invited contributors aged between 14 and 18 years who attend secondary school or college and had an Android smartphone.

PPI contributors were recruited from across the United Kingdom. A poster advertising the research was shared within schools and colleges. For recruitment from charities, emails were sent to adolescents within the target age range inviting them to contribute to PPI activities to improve a new sleep app.

PPI contributors could express their interest by scanning a QR code from the poster or following a link within each email. Prospective PPI contributors answered demographic Qualtrics survey questions (age, gender, and ethnicity), how often they had sleep problems, and their preferred method of engagement (one-to-one meeting in person or web-based discussion, web-based group discussions, or written comments). The levels of deprivation were estimated using contributors’ postcodes against the 2019 English indices of deprivation data. To ensure that adolescents from ethnically diverse and disadvantaged backgrounds were well represented, we prioritized input from volunteers from ethnic minority groups and more disadvantaged areas [65]. Over the course of a year, PPI contributors engaged in a variety of coproduction activities (Table 2), including identifying behaviors for the app to address; choosing the preferred name, appearance, and other key design elements; providing feedback on how prototype versions of the app could be improved; and trying out the app in real-life use and providing further feedback. PPI contributors shared their views according to their preferred method of contribution (Table 3). PPI contributions and activities are listed in Multimedia Appendix 1.

**Table 3:** Potential patient and public involvement contributors—preferred method of contribution (N=356).

Expressions of interest (January 2022-March 2022)—“Please select any of the ways you would be happy to talk to us”	Contributors, n (%)
Only written comments	219 (61.5)
Written comments	292 (82)
Web-based group discussion with other adolescents	65 (18.3)
One-to-one web-based meeting (eg, via Zoom)	63 (17.7)

### Phase 2: Intervention Optimization

#### Web-Based Think-Aloud Interviews

We invited contributors to provide detailed feedback on every aspect of the prototype app as it was developed using think-aloud interviews as an opportunity to identify barriers to engagement and feasibility and suggest improvements. PPI contributors met with a researcher via Microsoft Teams (Microsoft Corp) or Zoom (Zoom Video Communications) and provided verbal consent to take part in a one-to-one think-aloud interview. Contributors were given a web address and instructions on how to view Sleep Solved as it would appear on a mobile device. PPI contributors were encouraged to verbalize their immediate thoughts and feelings when interacting with Sleep Solved to provide as much information as possible about their impressions of the app and overall user experience [54]. Contributors were prompted for further information if required—such as “What are you thinking now?” or “What made you choose that option?”—and were encouraged to suggest ways of improving it (see Multimedia Appendix 2 for the topic guide). Interviews were audio recorded and transcribed verbatim. PPI contributors received a £22 (US $28.56) digital voucher for each hour of participation to thank them for their input after taking part in a think-aloud interview. All contributors who provided feedback via a Qualtrics survey were entered into a prize draw to receive a £50 (US $64.92) digital voucher. A versatile multiretailer voucher was carefully chosen by the research team in light of the cost-of-living crisis so that it could be used by contributors to purchase essential items such as food and toiletries. From PPI activity workshops 1 and 2, a total of 4 participants (n=2, 50% male; n=1, 25% female; and n=1, 25% genderfluid) also took part in the think-aloud sessions.

All feedback and suggestions from PPI contributors’ think-aloud interviews were collated in a table of changes (Multimedia Appendix 3). This was used directly as a basis for informing intervention changes rather than being subjected to analysis methods. Simple changes (such as clarifying text or rewriting a section that a participant found difficult to understand) would be made directly. Substantial changes were only made if they met the following criteria: (1) the proposed change was suggested by several PPI contributors, (2) the proposed change was likely to have an impact on users’ behavior, and (3) the proposed change aligned with the guiding principles for the intervention [54].

Once changes had been made, we obtained additional feedback from participants regarding the updated version of Sleep Solved in further think-aloud interviews [66,67]. The table of changes process continued until no new important suggested changes were raised in feedback from think-aloud interviews [66].

#### Sleep Solved Real-Life Testing: Think-Aloud Interviews

A selection of our PPI contributors was invited to trial the app for a period of 1 week after taking part in an initial think-aloud interview. After their trial, they were invited for a second think-aloud interview. PPI contributors were encouraged to follow the advice provided and keep a sleep diary for their personal reference during the follow-up interview (Multimedia Appendix 4).

#### Sleep Solved Real-Life Testing: Web-Based Workshops

The next stage of optimizing Sleep Solved started as soon as the “Must Have” changes were completed. Workshop attendees were contributors from the McPin Foundation’s Young People’s Network. All contributors were invited to use Sleep Solved for 1 week. Contributors with Android smartphones were also invited to use Phone Downtime alongside Sleep Solved. All were given a sleep diary checklist to complete and provide feedback about their experiences (Multimedia Appendix 5). A group workshop covered contributors’ experiences of engaging with every aspect of the app. Contributors were asked about elements of Sleep Solved that they liked or found useful and those that they did not like or found unhelpful or that did not work as expected. Questions explored contributors’ own sleep experiences and factors that helped them sleep well. The workshops were documented by group facilitators, with any suggested changes noted in the table of changes. A number of users created and recorded personal accounts of how they had benefited for other app users to listen to.

### Ethical Considerations

Ethics approval for the think-aloud interviews was granted by the University of Bristol School of Psychological Science Student Research Ethics Committee (ethics approval code 13084). PPI contributors provided verbal informed consent to take part in a one-to-one think-aloud interview. All data were anonymized, and all identifying information was removed. In compensation for their time, PPI contributors were offered a multiretailer digital voucher of £22 (US $28.56) for each hour of participation after taking part in a think-aloud interview. All contributors who provided feedback via a Qualtrics survey were entered into a prize draw to receive a £50 (US $64.92) digital voucher." (I've included the ethics approval statement that was there previously.)

## Results

### Overview

An overview of the Sleep Solved content is provided in Textbox 1.

**An overview of the Sleep Solved intervention content.**

**How can Sleep Solved help me sleep better?**

- Introduction to Sleep Solved: types of sleep problems, how sleeping better will help the user, and who made the app
- Explanation of the Phone Downtime app and link to download in Google Play

**How can less time in bed help me sleep better?**

- 3 kinds of sleep (deep, dreaming, and light)
- 5 to 6 hours and deep and dreaming sleep is what the brain needs. A lot of time in bed leads to additional light sleep—which the brain does not need as much.
- If the user does not sleep well one night, they will sleep better another night.
- Pop-up explaining the “point” of light sleep
- Explanation of why spending time in bed during the day can make it harder to fall asleep at night

**Why could sleeping in make me feel bad?**

- Explanation of the purpose of cortisol and its role in the sleep-wake cycle
- Graphs to evidence how irregular wake-up times impact cortisol release and how this, in turn, can impact mood
- Pop-up with tips to avoid napping impacting sleep (≤20 min, before 3 PM, and with the curtains open) and an explanation of why

**How can I stop worrying in bed?**

- Examples of common bedtime worries and explanation that this trains the brain to worry in bed
- Getting up after 20 min if unable to sleep and going back to bed when they feel sleepy
- Tips for calming activities to engage in instead
- Reiteration that, if they do not sleep well one night, they will sleep better another night
- Pop-up—tips on calming the mind before bed
- List of tips for calming the mind in bed

**What to do to sleep better?**

- The 1-week challenge; users are encouraged to try the 3 “sleep hacks” (sleep hygiene behaviors) for 1 week—getting up at the same time every day, helping their brain calm down before bed, and getting up after 20 min if unable to sleep.
- Pop-ups take the users to previous pages to remind them of the science behind the advice.
- Tips on how to make the “sleep hacks” easier—choosing what they want to do before they go to bed, telling friends and family about the challenge for help and support, and putting their phone out of reach so they will be unable to check it
- Normalization of tiredness and reiteration of napping rules
- Voice recordings (including transcripts) of adolescents explaining how they made the hacks work for them
- Link to personal reminders and diary
- Link to Sleep Healthy Using the Internet

**Track my progress and earn stars (added as a result of patient and public involvement feedback)**

- Optional reward system—switch to turn weekly challenge on or off
- Each day, the user selects whether they used the sleep hacks, partially used the sleep hacks, or did not use the sleep hacks the night before.
- If they used or partially used the sleep hacks, they earn a blue star.
- If the user collects 5 blue stars in a week, they earn a gold star.

**What other things could stop me sleeping well?**

- Problem and solution pairs explaining the science behind how various lifestyle factors (eg, caffeine, vaping, and social media) can impact sleep and offering harm reduction solutions

**Links to help the brain calm down**

- Links to calming sounds, mindfulness and meditation resources

**User journey**

- The user is presented with a splash screen with an animated Sleep Solved logo.
- Each key element is unlocked in a linear sequence once all pages (besides the optional pop-ups) of the previous element have been viewed.
- Element G—“What other things could stop me sleeping well?”—is unlocked 3 weeks after the user downloads Sleep Solved. Early access to this section is unlocked if the user earns 2 gold stars (see element F).
- The user can navigate back to the main menu at any stage using the hamburger menu.

### Phase 1: Intervention Development

#### Sleep Solved Ideas and Concept Generation

PPI contributors from the Bristol YPAG felt that the 3 ideas presented to them were novel. Many were keen for the app to be “soothing” and “simple,” with “noises that help you get to sleep” when feeling worried. These suggestions were implemented through a calming choice of colors; simple gestural app navigation; and the inclusion of “Links to help your brain calm down,” such as music and meditation sessions. The “How can I stop worrying in bed?” section gave ideas on calming activities.

Contributors expressed an interest in how phone use, food, and drink can affect sleep. These questions were addressed in the “What other things could stop me sleeping well?” section, which presents problems (how food, drink, and nicotine can affect sleep) with solutions (what users can do instead). How phone use can affect sleep at night was covered in the “How long should I try to get to sleep?” section, where users are advised to “avoid bright lights such as phone screens. Bright light can make your brain think it’s time to wake up!”

#### Sleep Solved Ideas: Name and Design

Results from the first Qualtrics survey helped create the overall look and feel of Sleep Solved. PPI contributors gave feedback on five different elements:

1. Potential app names: of the 7 options, “Sleep Solved” was chosen as the preferred name.
2. Color palettes: a black, opaline green, deep cream, and pale roundel blue color scheme was selected, with an average “like” score of 71% (SD 20.72%). Contributors shared that these colors were “simple,” evoking thoughts of “the sea” and “spring awakening.”
3. Logos: a bold sans-serif logo in all capital letters was chosen, with an average “like” score of 69% (SD 10.63%). Contributors liked the “bright colours” and readability.
4. Design themes: dark navy was chosen, with cartoonlike graphics in lime green, blue, purple, and orange. Praise was given for the “colourful,” “clear” design, which was “easy to find.”
5. Sleep Well recruitment materials: changes were made to the poster to make the fonts easier to read and the features of the app clearer.

#### New Theme Designs for Sleep Solved

On the basis of the feedback from the first Qualtrics survey, 2 potential app themes were designed by PIP Creative. Theme 1 was chosen in the second Qualtrics survey with an average “like” score of 68%. Contributors liked the “sleepy colour theme” and dark colors, which “connotate sleep”; “contrasting colours are cool, it’s very appealing.” They preferred brightly colored icons as these “draw in my attention.” Less praise was given to theme 2, a softer theme with rounded dark purple and navy iconography: “it’s not as vibrant and interesting” and “too dull, doesn’t draw my attention.”

#### New Logo Designs for Sleep Solved

In total, 4 logos were presented to PPI contributors in the third Qualtrics survey. PPI contributors found a colorful, bold logo made of interconnecting shapes “difficult to read...because of the colour scheme.” A bold sans-serif logo was chosen in white and yellow. The bright colors featured in a geometric fan shape next to the logo, making it “better [for] being colourful” but still easy to read.

#### Sleep Well Design Feedback

The results of the fourth Qualtrics survey are shown in Tables 4 and 5.

**Table 4:** An overview of the Sleep Solved intervention content.

Route	How likely are you to take part for 6 weeks? (0-100), mean (SD)
1—Phone Downtime for 6 weeks, then access to Sleep Solved for 6 weeks, then access to SHUTi^a^	64.3 (26.1)
2—Phone Downtime and Sleep Solved for 6 weeks, then access to SHUTi	63.3 (25.4)
3—Access to all 3 components right away	55.7 (27.5)

^a^SHUTi: Sleep Healthy Using the Internet.

**Table 5:** An overview of patient and public involvement contributors’ preferred Sleep Solved intervention content (n=23).

	First place		Second place			Third place	
	Values, n (%)	Values, mean (SD)	Values, n (%)	Values, mean (SD)	Values, n (%)		Values, mean (SD)

Route 1	7 (30)	2.09 (0.83)	7 (30)	2.09 (0.83)	9 (39)		2.09 (0.83)
Route 2	7 (30)	1.70 (0.55)	14 (60)	1.70 (0.55)	1 (4)		1.70 (0.55)
Route 3	9 (39)	2.22 (0.93)	2 (8)	2.22 (0.93)	13 (56)		2.22 (0.93)

When asked to rank their preferred options for phasing the steps of the intervention, there was no clear leader. Route 3 was ranked as contributors’ first choice 9 times but was ranked in last place by 13 PPI contributors. As route 2 was ranked second by most people (14/23, 60%), this was chosen as a compromise as very few (2/23, 8%) ranked this as their last-placed option compared to route 3 (13/23, 56%). PPI contributors shared that they felt that route 2 was the most time-efficient, logical option:

I think [Route 2 is] the best way for people to be able to see how the app is helping their sleep schedule which encourages them to keep using it...then they can determine whether they need the SHUTi app or not.

PPI contributor; female; aged 17 years; White British ethnicity

Phone Downtime and Sleep Solved don’t require much effort so they can be both done at the same time.

PPI contributor; male; aged 14 years; White British ethnicity

[Route 2] is less time consuming.

PPI contributor; nonbinary person; aged 18 years; White British ethnicity

### Phase 2: Intervention Optimization

#### Web-Based Think-Aloud Interviews

Results from the think-aloud interviews were largely positive, with praise for the clear, easily understood content and ease of functionality:

I like the way it’s broken up into sections.

JK02

I think one of my favourite parts about it is the explanations behind a lot of it. I like knowing kind of why I’m doing something rather than just saying like this person says it works.

JK06

“Science based”—makes it more trustworthy, I’m hopeful.

JK05

It’s nice to know, there’s various ways that you can improve your sleep, there’s not just one way, and that it’s okay if one doesn’t work for you because there’s always another and that you can start off easy and then like, build up.

JK03

Nevertheless, the interviews identified numerous small but important ways in which the intervention could be improved. A summary of the think-aloud feedback and the changes implemented can be found in Table 6. Examples from the table of changes can be found in Multimedia Appendix 2. Less positive responses focused on areas in which users found information difficult to understand as it was originally presented or fonts were hard to read.

**Table 6:** Summary of changes to Sleep Solved as a result of think-aloud interview feedback.

Section of Sleep Solved and problem identified		Changes made
**Overall design**		
	“The contrast of the light blue text to the white background on highlighted words makes it difficult to read—I have dyslexia. Prefer the white text on a dark background.” [JK04] “The terms ‘anxiety’ and ‘depression’ could be seen as quite negative or serious terms.” [GT01]	Light blue font was changed to darker blue font throughout to be easier to read. We retained the use of bold and italic for emphasis. References to anxiety or depression were changed to “worried” or “low mood” throughout to reduce any negative connotations.
**How can Sleep Solved help me sleep better?**		
	“Who made this app?”: “Might be good to include more information on why these people are interested in contributing to the app.” [JK02] “This section is unnecessary; I don’t care who made the app—I trust if you say it’s backed by science.” [MJ02]	The “Who made this app” page was changed to an optional popout: “Who made this app? Tap here.” Photos introducing each team member were amended to a brief paragraph of text recognizing the valuable contributions of hundreds of adolescents and providing links to partner charities.
	“Get good marks—isn’t clear that you’re talking about school. ‘Do better in school’ would be better.” [MJ01] “Improve your grades would be better.” [MJ02]	References to improving grades were removed as it was felt that this may not be an achievable goal or may not be a priority for all the adolescents using the app.
**Why could sleeping in make me feel bad?**		
	“The order of the information is hard to understand.”[MJ01]	The text on the final card, “Getting up at the same time every day trains your brain to release cortisol at the right time,” was moved to the first slider. The “if you sleep late your cortisol levels peak late” text was combined with “this may make you feel tired and sleepy when you have to get up earlier” to improve understanding.
	“Is it OK to nap when I’m tired?”: “Why do you have to nap before 3pm?” [GS01 and JK06] “Why 20 minutes?” [JK06]	Explanatory text was added to clarify that a 20-min nap would prevent users from sleeping too long or too deeply. Furthermore, we provided evidence of the science behind this recommendation—that, if users sleep late in the day, their brain will release cortisol on waking, making them feel more awake and making it more difficult to sleep later that night.
**How can I stop worrying in bed?**		
	“My brain never really stops thinking, maybe tell us how to stop thinking.” [JK07]	Text changed from “thinking” to “worrying”: “Lying awake at night, you might start to worry about: how tired you will feel in the morning, all the other worrying things in your life. This trains your brain to worry in bed instead of sleeping!”
**What to do to sleep better?**		
	“The title, ‘What to do to sleep better’ does not make it clear that this is the one-week challenge.” [MJ01]	A front page was added stating the following: “This is your one-week challenge! Try these sleep hacks for 1 week!”
**Track my progress and earn stars**		
	Caffeine—problem: “An exhaustive list of everything that contains caffeine would be helpful. This could be presented as a mind-map to make it more visually appealing.” [MJ06] Caffeine—graph: “Would be good to indicate the 10hr period somewhere on the graph.” [MJ06] “The sun/moon/cloud symbols don’t really add anything—they’re confusing.” [MJ06]	A list of all drinks and foods containing caffeine was added as a pop-up section. Changes were made to a graph depicting caffeine levels in the body over time. Consumption of 2 caffeinated drinks was shown over the course of a day, and more accurate timings were added to represent how caffeine levels in the body change over time.
	Food—solution: “It would be good to have a more extensive list of the things that you can eat.” [MJ06]	More foods were added to the list.

#### Sleep Solved Real-Life Testing: Think-Aloud Interviews

##### Overview

Table 7 provides an overview of each PPI contributor’s sleep hack use during their 1-week trial of the Sleep Solved app before their second think-aloud interview and the perceived strengths and limitations of each hack.

**Table 7:** Feedback on Sleep Solved use.

Unique user identifier	Use pattern	Strengths	Limitations
C1	Hack 1^a^	Hack 1: going to sleep earlier Hack 1: feeling less tired and stressed during the day	—^b^
C2	Hack 1 Hack 2^c^	Hack 1: falling asleep more quickly Hack 2: feeling more awake in the morning Hack 2: increased productivity and focus Hack 2: falling asleep faster	Hack 1: found it difficult to stick to it on weekends Hack 2: time-consuming during examination periods
C3	Hack 1 Hack 2 Hack 3^d^	Hack 1: feeling more energized in the morning Hack 2: reduced worried thoughts and falling asleep faster Hack 3: falling asleep more easily	Hack 1: not motivated to maintain the behavior in the long term Hack 2: did not maintain the behaviors in the long term; enjoys using their phone to watch videos or message friends before bed
C4	Hack 1 Hack 2 AN^e^	Hack 1: feeling more rested Hack 2: falling asleep more quickly AN: falling asleep earlier	Hack 1: challenging to follow (part-time job with evening shifts) AN: hard to maintain when very tired and not busy
C5	Hack 1 Hack 2 Hack 3	Hack 1: falling asleep earlier, napping less during the day, and waking up less during the night	Hack 1: still sleeping lightly Hacks 1 and 2: did not improve sleep
C6	Hack 1 Hack 2 Hack 3	Hack 1; falling asleep earlier, sleeping more deeply, and feeling more alert and refreshed in the mornings Hack 2: reduced anxiety before bed Hack 3: falling asleep more quickly	Hacks 1 and 2: would be hard to maintain when traveling and staying at friends’ houses Hack 3: difficult to maintain during the school holidays
C7	Hack 2 Hack 3	Hack 2: falling asleep earlier and more quickly	Hack 3: did not improve sleep

^a^Getting up at the same time every day.

^b^No limitations suggested.

^c^Calming your brain.

^d^Less time in bed.

^e^AN: avoiding napping.

##### Set an Alarm to Get Up at the Same Time Every Day (Hack 1)

PPI contributors reported great variability in their sleep and wake times before trying hack 1, largely due to varying class schedules. Many of those involved reported a notable change in their sleep schedules after trying this hack: falling asleep and rising earlier. However, hack 1 was not feasible for PPI contributors who had to work late-night shifts and, therefore, had a variable sleep schedule. Within the socioecological model, this would correspond to the work, school, and college moderator, which recognizes the impact of variable school, college, and work start times on sleep, particularly in children and adolescents [57,68]. Several PPI contributors described needing motivation to continue the sleep hack on the weekends or during the holidays. This was particularly relevant when taking examinations as they were more likely to try to “catch-up” on sleep on the weekends and were more reluctant to get up at the same time as on weekdays.

##### Help Your Brain Calm Down Before You Go to Bed: Do Not Do Anything Exciting or Stressful (Hack 2)

Relaxation strategies were perceived as positive by most PPI contributors, finding that this advice helped them reduce their anxious thoughts and fall asleep faster. Calming activities described by contributors included reading, watching television, listening to relaxing music, and ensuring that they stopped homework or revision activities with enough time to “wind down” before bed. Others explained how stopping the use of social media apps before bed had really helped improve their sleep, feeling more “refreshed” on waking. However, a minority found it difficult not to use their phones in the hour before bed, and they continued to message their friends, use social media, and watch videos. In the socioecological model and our logic model, this is represented by the social networks moderator—the friends, acquaintances, and networks that contribute to roles, expectations, beliefs, and behaviors regarding sleep [57].

##### If You Do Not Get to Sleep After 20 Minutes, Get Up and Do Something Calm Until You Are Sleepy (Hack 3)

Despite initial worries that following this hack would be “impossible,” contributors reported finding it easier to fall asleep on returning to bed instead of their usual practice of using their phone or lying awake unable to sleep. Although perceived as initially challenging, after a few days of practice, many could follow the hack, and their sleep latency improved over time. For a minority, this was their favorite sleep hack, and one user described counting to their 20-minute target as so restful that they fell asleep. PPI contributors reported that spending less time in bed helped reduce the overthinking and associated anxiety that they experienced. However, one contributor had concerns that getting up and moving around may disturb other people in their household.

##### Overall Acceptability

Sleep Solved was deemed acceptable by the PPI contributors. They were appreciative of the range of text formats, simple language, and the use of bullet points to break up information, as well as the use of bright imagery. They described the interactive “slider” navigation elements as “engaging,” “fun,” and “encouraging.” Users praised the science-based content, which provided them with simple yet clear explanations of the reasoning behind the (seemingly contradictory) behaviors to sleep better, such as spending less time in bed. The use of choice architecture (the framing of the advice given as an option, consistent with the guiding principles) was well received. Contributors were given the benefits of the desired behavior compared to the consequences of undesired behaviors. By not telling users which behavior they “should” be doing, many were keen to follow the behavior when the benefits and reasoning for the advice were well explained:

I think it explaining that little bit more makes you feel more kind of like it’s a good thing, because I’m more aware of what’s going on rather than just this is what I’m being told it’s going on in my brain, but it makes you feel more kind of knowledgeable in it. It feels very personalized because I can kind of relate to that, that is exactly...how I feel.

PPI contributor C4; female; aged 18 years; sleep problems 2-3 times per week

Being given clear reasons for following the suggested behaviors in a particular way was similarly well received by users:

[The science-based approach] it’s informative in a non-patronising way. If you’re patronised, then you’re not going to listen...Like if your parents tell you to do something, they’re describing [it to] you, but if you know why you should do it, you’re more likely to be doing it.

PPI contributor C1; female; aged 17 years; sleep problems 2-3 times per week

It’s a bit humorous as well, which is quite interesting. Rather than...just telling you what to do and what not to do.

PPI contributor C2; female; aged 16 years; sleep problems 2-3 times per week

##### Web-Based Workshop 1: Sleep Solved Real-Life Testing

Feedback from these users was positive. PPI contributors considered the bullet points, visual aids, and graphs to be “helpful for understanding.”

#### Set an Alarm to Get Up at the Same Time Every Day (Hack 1)

This hack was cited as being harder to stick with, particularly on the weekends or during holidays. PPI contributors felt that not having to get up for school or college made their sleep “less disciplined” (M1) as on these days they were able to procrastinate, putting off getting up and out of bed for longer (M2 and M3). However, one contributor liked the fact that, by charging their phone at the other side of the room, they could not be tempted to check on it; they felt that this advice was very helpful (M2).

#### Help Your Brain Calm Down Before You Go to Bed: Do Not Do Anything Exciting or Stressful (Hack 2)

All PPI contributors liked the concept of taking part in calming activities before bed, sharing that they felt “more refreshed when I wake up” (M3). Stopping exciting or stressful activities such as homework, revising for examinations (M3), or watching Netflix or TikTok before bed (M7) was “really helpful” (M7) and helped them sleep (M2). One PPI contributor shared that this was their examination season, so they were keen to get as much sleep as possible (M1).

#### If You Do Not Get to Sleep After 20 Minutes, Get Up and Do Something Calm Until You Are Sleepy (Hack 3)

Although this sleep hack was the favorite of some PPI contributors (M7), others worried that they may disturb other people in their family when getting out of bed or moving around (M2), which, when considering the socioecological model, recognizes the impact of moderators such as family, socioeconomic status, and environment on sleep [57]. For some, counting the 20 minutes until it was time to sleep was like “counting sheep,” and they found that they fell asleep anyway (M3).

#### Web-Based Workshop 2: Phone Downtime Testing

##### Overview

PPI contributors also tested the Phone Downtime app for 1 week, which was an app that was included for Android users of Sleep Solved. Users were encouraged to set the time when they usually went to sleep and woke up. The Phone Downtime app enabled users to tell when they were using their phone during the time they planned to be asleep (during a time window preset by the user).

PPI contributors liked being able to choose their own goals and sleep times and the fact that working days were flexible to accommodate part-time jobs on different days. A new awareness of for just how long they were using their phones at night was welcome as it helped them better schedule their bedtime.

##### The Need for Interaction

A large proportion of PPI contributors were keen on the addition of a progress chart, journal, or sleep diary. Many expressed a wish to track and measure their sleep behavior changes over time. In its initial prototype stage, adolescents did not feel that there was much to attract them to Sleep Solved once they had completed all the sections. They suggested adding an interactive reward system (eg, where they could collect points and monitor their progress over time).

As a result of contributor feedback, an optional reward system was added to Sleep Solved. Under a new section, “Track my progress and earn stars,” users can activate their rewards using a toggle switch to turn their weekly challenge on or off. Each day, a pop-up asks the user whether they (1) used the sleep hacks, (2) partially used the sleep hacks, or (3) did not use the sleep hacks the night before. If they used or partially used the sleep hacks, they earn a blue star. If the user collects 5 blue stars in a week, they earn a gold star. Collecting 2 gold stars enables early access to supplementary content within the app, which provides additional information about the influence of behaviors such as vaping, drinking caffeine, and diet on sleep.

While the need for adolescents to continue to return to the app was not a requirement for effective engagement [69], similar sleep behavior apps have also used elements of gamification and reward systems. These include users tracking their sleep for 3 nights out of 7 to progress to their next “belt” in Sleep Ninja [45], gift cards for users of Sleepio, a digital app to improve sleep behavior using internet-based cognitive behavioral therapy [70], and monetary rewards for children who met their predefined sleep goals [71]. In an exploration of young adults’ perspectives on gamification in health apps, rewards such as badges and progress indicators were viewed more positively than self-rewards [72]. Young adults with depression and anxiety have expressed a preference for self-tracking behaviors over time; gamified elements that rewarded progress toward their behavior “goals” increased their motivation to engage with the app and regularly track their behavior [73].

PPI contributors were keen to share their own sleep-related experiences for other adolescents who may use the app in the future. A total of 7 adolescents who participated in the workshops recorded relatable stories related to poor sleep and how Sleep Solved had helped them. A variety of ages, ethnicities, and regional accents were represented following the guiding principle to “ensure that role models and stories were relevant to diverse contexts and identities.” These were presented to app users as playable audio clips.

## Discussion

### Principal Findings

This study used the person-based approach to design and optimize the first step of a 3-stage, app-based intervention aimed at improving sleep-related behaviors in adolescents aged 14 to 18 years. Sleep Solved was considered acceptable, feasible, and easy to use by PPI contributors, including those from underrepresented and diverse backgrounds.

This study highlights that offering varied means of contributing to intervention coproduction is helpful in terms of maximizing the speed of the co-creation process and the diversity of the sample of PPI contributors. It also confirms that, for advice to be engaging for adolescents, it is essential that it is perceived as trustworthy and novel and framed as a choice. A key finding from this study was the importance of offering our PPI contributors varied methods of contribution. This allowed contributors to express feedback in the way in which they felt most comfortable, resulting in diverse and complementary insights.

Web-based surveys were the most popular contribution method and allowed us to purposively recruit adolescents from low-income communities. Web-based surveys facilitated quick feedback on multiple-choice decisions, such as the app name, theme colors, and pathway options for the study. The second most popular contribution method was to take part in a web-based, one-to-one, think-aloud interview. These interviews provided in-depth feedback, allowing us to iteratively optimize the app over a 7-month period.

In comparison to previous research, this study used a 3-stage, app-based intervention, incorporating a co-created first stage followed by CBTi (SHUTi) and mental health support (Bite Back). Levenson et al [74] also designed adolescent sleep interventions with stakeholder input and involvement in a series of 3 focus groups with young adults, adolescents, and health care professionals with experience working with adolescents. These focus groups informed the development of a clinic-based sleep intervention for adolescents aged 13 to 15 years. Similarly, the “Momentum” intervention for young people with insomnia and mental health difficulties was co-created with young people aged 7 to 17 years, with participants encouraged to give feedback on the “look and feel.” As advised in the recommendations from the Momentum study, Sleep Well has features that encourage interaction, such as games, videos, interactive elements, quizzes, and rewards.

As with our SHUTi insomnia intervention [51], Palermo et al [75] also used CBTi to develop a 4-session CBTi intervention for adolescents with insomnia and co-occurring mental health conditions. Participants were encouraged to complete sleep diaries during treatment. The results indicated a high level of acceptability and feasibility, with high compliance; 85% (n=34) completed all 6 intervention sessions. Later pilot-testing indicated that the CBTi intervention was associated with improvements in insomnia symptoms, sleep quality, and sleep onset latency.

Focus group and workshop contributors, who were recruited from existing networks such as the Bristol YPAG and the McPin Foundation, were generally highly confident and motivated to engage with the study. They engaged thoroughly with the app over the course of a week, providing important suggestions, and were sufficiently confident to record their personal experiences for inclusion in the app.

### Strengths

In line with our guiding principle to ensure that Sleep Solved was perceived as interesting, trustworthy, and novel, our results highlighted that PPI contributors particularly valued the accessible presentation of the science-based advice in Sleep Solved. The perception of the advice as novel was important as adolescents have been shown to have a very low tolerance for behavior change interventions that they perceive as dull or boring [63,64]. The perception of the advice as trustworthy may be a key distinguishing feature of Sleep Solved; in a review of the 76 sleep-related health apps available on Google Play, less than 1 in 3 contained evidence to support their claims [60]. In line with our guiding principle to ensure that advice is framed as a choice, PPI contributors particularly appreciated not feeling “patronized” by the advice in Sleep Solved.

The key strength of this study is that our intervention was coproduced from start to finish, with quality in-depth, varied, and iterative feedback from PPI contributors. The role of the peer researcher (MHJ) in building ongoing, trusting relationships with contributors was a key feature of this success.

### Limitations

One major limitation is the representativeness of our PPI contributors. Of those invited to share their feedback, only a small proportion volunteered to contribute to co-design. Therefore, it is likely that our contributors were a more highly motivated subsection of our target demographic, and it is possible that their recommendations may not always represent the views of less motivated adolescents.

In addition, a significantly smaller number of adolescents aged 14 to 15 years volunteered to share their views in think-aloud interviews and group workshops. It is possible that recommendations from those who did not wish to engage with us in more depth may have differed from the suggestions of the adolescents aged 16 to 18 years who helped us develop the app.

### Future Work

To foster a more representative PPI sample, forming early relationships with individuals from the relevant community with whom recruitment procedures can be coproduced [69] will likely be beneficial. The formation of these relationships may be made easier by having a more diverse research team [70].

### Conclusions

In conclusion, offering multiple methods of providing feedback allows for the collection of holistic feedback from a diverse range of contributors. This feedback resulted in an app viewed as acceptable and engaging by the adolescents who have tried it to date. Further evaluation is needed to determine the feasibility and acceptability of the intervention in a larger group of adolescents, and future research should explore ways of engaging hard-to-reach young people in intervention development research.

## Appendix

Multimedia Appendix 1 Patient and public involvement (PPI) activities—engagement activities with our PPI contributors in chronological order.

Multimedia Appendix 2 An overview of think-aloud questions and prompts asked to patient and public involvement contributors when trialing the prototype Sleep Solved intervention.

Multimedia Appendix 3 Examples from the table of changes.

Multimedia Appendix 4 The Sleep Hacks Diary used in think-aloud interview user testing.

Multimedia Appendix 5 A prototype Sleep Solved app feedback template for patient and public involvement contributors to complete notes and feedback about their experiences.

## Abbreviations

CBTi: cognitive behavioral therapy for insomnia
DOZE: Delivering Online Zzz’s With Empirical Support
PPI: patient and public involvement
YPAG: Young People’s Advisory Group
