# Supplementary material for: Using the Person-Based Approach to Co-Create and Optimize an App-Based Intervention to Support Better Sleep for Adolescents in the United Kingdom: Mixed Methods Study
Source: JMIR Hum Factors. 2024 Oct 31;11:e63341. doi: 10.2196/63341 (PMC11565086; doi:10.2196/63341)
Supplement: Multimedia Appendix 1 [file humanfactors_v11i1e63341_app1.docx]

**Multimedia Appendix 1:** Patient and public involvement (PPI) activities—engagement activities with our PPI contributors in chronological order.

| Focus | Method of contribution | Organization they were recruited from | PPI contributors | Outcome |
| --- | --- | --- | --- | --- |
| Sleep Solved ideas and concept generation | Web-based focus group (September 8, 2022) | Bristol YPAG^a^ | - 6 PPI contributors: 3 women and 3 men - Age range: 14-17 years - Recruited from Bristol YPAG - Demographic data were not sourced | - PPI contributors suggested ideas and concepts related to sleep and well-being support, as well as possible sleep behaviors that the app should address. |
| Sleep Solved name, design, and study recruitment materials | Qualtrics survey 1 (November 2, 2022) | The Association of Colleges and E-ACT^b^ multi-academy trust | - 5 PPI contributors: 4 women and 1 who reported “prefer not to say” - Age range: 14-17 years - Half were from the 10% most deprived areas in the United Kingdom | - PPI contributors expressed their preferences regarding examples of app names, color palettes, logos, and themes. These examples were based on other successful designs aimed at adolescents. They provided feedback to improve the readability and acceptability of the Sleep Well study advertisement poster and Sleep Well participant information sheet. |
| New theme designs for Sleep Solved | Qualtrics survey 2 (November 28, 2022-December 2, 2022) | The Association of Colleges and E-ACT multi-academy trust | - 7 PPI contributors: 5 women and 2 men - 6 were of White British ethnicity and 1 was of Sri-Lankan or Sinhalese ethnicity - All aged 16 years - From the 30%-40% least deprived areas in the United Kingdom | - PPI contributors expressed their preference between 2 mobile app design themes illustrated by PIP Creative. These themes were ranked by PPI contributors, and they provided their reasons for the rankings. |
| New logo designs for Sleep Solved | Qualtrics survey 3 (February 2, 2023-February 14, 2023) | The Association of Colleges and E-ACT multi-academy trust | - 9 PPI contributors: all women - 7 were of White British ethnicity and 1 was of White and Black African ethnicity - Age range: 16-17 years - 6 of the 9 were from the 20%-50% most deprived areas in the United Kingdom | - PPI contributors gave feedback and suggestions on 4 possible logo options for Sleep Solved that were designed by PIP Creative. |
| Sleep Solved real-life testing | Think-aloud interviews before and after a 1-week trial of the app (January 16, 2023-February 13, 2023) | The Association of Colleges, E-ACT multi-academy trust, and Off The Record | - 7 PPI contributors tested the prototype app: 6 women and 1 nonbinary person - 6 were of White British ethnicity, and 1 user was of Middle Eastern ethnicity - Age range: 16-18 years - Levels of deprivation ranged from the 40% most deprived to the 20% least deprived areas - Most had self-identified sleep problems ranging from 2 to 3 times per week (n=5) to >4 times per week (n=1) | - PPI contributors tried out the Sleep Solved prototype for 1 week. They were then invited to participate in a think-aloud interview, during which they provided their feedback and recommendations. |
| Sleep Solved real-life testing | Think-aloud interviews while trialing the Sleep Solved prototype app (January 12, 2023-May 19, 2023) | The Association of Colleges, E-ACT multi-academy trust, and the McPin Foundation | - 12 PPI contributors tested the prototype app: 7 men, 4 women, and 1 genderfluid person - 1 was of White British ethnicity, 1 user was of Arab ethnicity, 1 was of African ethnicity, 1 was of African and Black Caribbean ethnicity, 1 was of White and Black Caribbean ethnicity, 1 was of Pakistani ethnicity, 1 was of South Asian ethnicity, 1 was of Middle Eastern ethnicity, and 1 preferred not to say - Age range: 16-18 years - Levels of deprivation ranged from the 30% most deprived to the 40% least deprived areas - Most had self-identified sleep problems ranging from 2 to 3 times per week (n=3) to >4 times per week (n=3) | - PPI contributors tried out the Sleep Solved prototype during their think-aloud interview. As they worked through the app, they provided their feedback and recommendations. |
| Sleep Solved real-life testing | Web-based workshop 1 (April 12, 2023) | The McPin foundation | - 9 users tested the prototype app: 5 young men and 4 women - 4 were of White British ethnicity, 1 was of Pakistani ethnicity, 1 was of Black Caribbean ethnicity, 1 was of Black African ethnicity, 1 was of Black British ethnicity, and 1 was of “Other Asian: Nepali” ethnicity - Age range: 15-18 years - 5 of the 9 were from the 10%-30% most deprived neighborhoods in the country | - PPI contributors tried out the Sleep Solved prototype for 1 week before this web-based workshop, during which they provided their feedback and recommendations. |
| Sleep Well study order and design feedback | Qualtrics survey 4 (April 24, 2023) | The Association of Colleges and E-ACT multi-academy trust | - 22 PPI contributors provided feedback: 15 women, 5 men, 1 genderfluid person, and 1 nonbinary person - 16 were of White British ethnicity, 1 was of White Irish ethnicity, 2 were of African ethnicity, 1 was of White and Black Caribbean ethnicity, 1 was of Pakistani ethnicity, 1 was of “Other White: European” ethnicity, and 1 was of “Other Asian: Nepali” ethnicity - Age range: 14-18 years - 13 were from the 10%-50% most deprived areas in the United Kingdom | - PPI contributors provided feedback on how they wanted the combination of the 4 components to be presented to them and in which order. |
| Phone Downtime real-life testing and design feedback | Web-based workshop 2 (July 25, 2023) | The McPin Foundation | - 9 PPI contributors: 5 men and 4 women - 4 were of White British ethnicity, 1 was of Pakistani ethnicity, 1 was of Black Caribbean ethnicity, 1 was of Black African ethnicity, 1 was of Black British ethnicity, and 1 was of “Other Asian: Nepali” ethnicity - Age range: 15-18 years - 5 were from the 10%-30% most deprived areas in the United Kingdom | - PPI contributors tested the Phone Downtime prototype app for 1 week. In total, 2 PPI contributors from workshop 1 contributed to workshop 2. |

^a^YPAG: Young People’s Advisory Group.
