# Supplementary material for: Using the Person-Based Approach to Co-Create and Optimize an App-Based Intervention to Support Better Sleep for Adolescents in the United Kingdom: Mixed Methods Study
Source: JMIR Hum Factors. 2024 Oct 31;11:e63341. doi: 10.2196/63341 (PMC11565086; doi:10.2196/63341)
Supplement: Multimedia Appendix 2 [file humanfactors_v11i1e63341_app2.docx]

**Multimedia Appendix 2:**An overview of think-aloud questions and prompts asked to PPI contributors when trialling the prototype Sleep Solved intervention.

**Part 1: Introduction**

1. Can you tell me why you were interested in being part of this study?

- Prompt: Can you share some reasons falling asleep is challenging for you (e.g. stress, noise)?

**Part 2: Think aloud with the Sleep Solved prototype**

1. As you go through each page of the Sleep Solved app, can you tell me what you think about it? Feel free to say out loud any thoughts that come to mind as you look through it.

Prompts:

- [on the first viewing of the Sleep Solved app Home screen] What are your first thoughts?
- What are you thinking now?
- What made you choose that option?
- What do you think about this message/advice/information?
- Can you tell me a bit more about why you think that?
- What is it you like/don’t like about that?
- Can you share with me more about why that is important to you?

**Part 3. Post think-aloud questions**

1. Overall, what do you think of the app?

Prompts:

- [If PPI contributor responds with thoughts about the style/function of the app] What do you think of the advice in the app?
- Can you tell me anything you found particularly good about the app or the advice?
- Can you tell me anything about the app and the advice it gave you were less keen on?

1. Can you share with me what do you think should be changed?

1. What do you think it would be like using the app in real life?

**Part 4: Closing**

1. Thank you for sharing your thoughts about this app with me. Do you have anything else you would like to share with me that we haven’t already covered?
