# Supplementary material for: Using the Person-Based Approach to Co-Create and Optimize an App-Based Intervention to Support Better Sleep for Adolescents in the United Kingdom: Mixed Methods Study
Source: JMIR Hum Factors. 2024 Oct 31;11:e63341. doi: 10.2196/63341 (PMC11565086; doi:10.2196/63341)
Supplement: Multimedia Appendix 3 [file humanfactors_v11i1e63341_app3.docx]

**Multimedia Appendix 3:** Examples from the Table of Changes.

| **Intervention Section** | **Negative Comments** | **Positive Comments** | **Possible change** | **Reason for change** | **Agreed change** | **MoScoW** |
| --- | --- | --- | --- | --- | --- | --- |
| **A. How can Sleep Solved help me sleep better?** | It would be better if you could click and find further descriptions for why sleeping better could help with thinking faster, feeling happier etc. | This is clear and easy to read.  I like the layout.  It's good to learn how improving sleep can help you.  This is motivating. | Include evidence-based claims to support these statements. | NREP, HLIT | No change as most people liked current format, adding text might make app less accessible | N/A |
| **B. How can less time in bed help me sleep better?** | A more thorough definition for cortisol than 'get up and go' would be useful |  | Add a more in-depth scientific explanation of cortisol for those interested | HLIT, NREP | No change  as only one high literacy person wanted more text | N/A |
| **C. Why could sleeping in make me feel bad?** | Why does it have to be before 3pm?  Why only 20 minutes – that’s really short. | This is interesting  This makes sense  I like this information  This is new information | Add more detail to explain science behind napping restrictions | IMP, REP, EAS | Add more detail to explain science behind napping restrictions | Should have |
| **D. How can I stop worrying in bed?** | “Don't worry about feeling tired the next day” comes across patronising, especially if you've chronically been struggling with your sleep | I find this reassuring. Would help me to not worry so much about feeling tired the next day.  I think this makes sense that you'll get more sleep the next day. | Direct to SHUTi sooner | NREP | No change as most people liked the text | N/A |
| **E. What to do to sleep better?** | It’s not immediately clear that this is the one-week challenge |  | Change the first page of this section to "This is your one-week sleep challenge!" | EAS, IMP | Change the first page of this section to "This is your one-week sleep challenge!" | Must have |
| **E. What to do to sleep better?** | When clicking on the recap buttons, it goes back to section C, so I have to click through two sections get back to where I was. |  | After closing recap, bring users back to the same page within section E. | IMP, REP | After closing recap, bring users back to the same page within section E | Must have |
| **F. Track my progress and earn stars** | It would be more motivating to add a calendar which shows you the week(s) ahead and all the stars you could collect | The idea of being able to unlock new content is really exciting  I think this would be really motivating | Add a calendar to progress tracking page | NREP | No change at present as only one person suggested, do not have time and resource to do now | Would like to have |
| **Change acronyms**: EAS: easy; IMP: important; REP: repetitive  **No change acronyms:** NREP: not repeated; NIMP: not important; HLIT: relevant to only highly literate users  MoScoW: Must have, Should have, Could have, Would like to have. | | | | | | |
