# Supplementary material for: Using the Person-Based Approach to Co-Create and Optimize an App-Based Intervention to Support Better Sleep for Adolescents in the United Kingdom: Mixed Methods Study
Source: JMIR Hum Factors. 2024 Oct 31;11:e63341. doi: 10.2196/63341 (PMC11565086; doi:10.2196/63341)
Supplement: Multimedia Appendix 4 [file humanfactors_v11i1e63341_app4.docx]

**Multimedia Appendix 4**: The Sleep Hacks Diary used in think aloud interview user testing.

| **_____________’s Sleep Hacks Diary**  Try these sleep hacks for one week! Tick the box if you tried the sleep hack. | | | | | | | |
| --- | --- | --- | --- | --- | --- | --- | --- |
| **Day** | **1** | **2** | **3** | **4** | **5** | **6** | **7** |
| **Date** |  |  |  |  |  |  |  |
| **HACK 1: Set an alarm to get up at the same time everyday** | 🞏 | 🞏 | 🞏 | 🞏 | 🞏 | 🞏 | 🞏 |
| **HACK 2: Help your brain calm down before you go to bed - don’t do anything exciting or stressful** | 🞏 | 🞏 | 🞏 | 🞏 | 🞏 | 🞏 | 🞏 |
| **HACK 3: If you don’t get to sleep after 20 minutes, get up and do something calm until you are sleepy** | 🞏 | 🞏 | 🞏 | 🞏 | 🞏 | 🞏 | 🞏 |
| **Notes:** |  |  |  |  |  |  |  |
