# Supplementary material for: Using the Person-Based Approach to Co-Create and Optimize an App-Based Intervention to Support Better Sleep for Adolescents in the United Kingdom: Mixed Methods Study
Source: JMIR Hum Factors. 2024 Oct 31;11:e63341. doi: 10.2196/63341 (PMC11565086; doi:10.2196/63341)
Supplement: Multimedia Appendix 5 [file humanfactors_v11i1e63341_app5.docx]

**Multimedia Appendix 5**: A prototype Sleep Solved app feedback template for PPI contributors to complete notes and feedback about their experiences.

| This diary is for you to write down your thoughts about Sleep Solved as you use it.  You can write notes in any way that you want. This is just a guide to help you remember your views and ideas for when we chat as a group on Zoom.  Good or bad, we want you to be totally honest about what you think about Sleep Solved. | | | | | |
| --- | --- | --- | --- | --- | --- |
| **Sleep Hack** | **What did you like / find helpful?** 👍 | **What did you dislike / find unhelpful?** 👎 | **Did anything make it harder to use?** 🤔 | **Did anything make it easier to use?** 😀 | **Is there anything else you would like to note down? ❓** |
| Getting up at the same time everyday |  |  |  |  |  |
| Calming your brain |  |  |  |  |  |
| Less time in bed |  |  |  |  |  |
| Overall: Design (i.e., the colours, images, buttons available to you) |  |  |  |  |  |
| Overall: Content (i.e., the wording, what was included or not included) |  |  |  |  |  |
